# Dislocation mechanisms and 3D twin architectures generate exceptional strength-ductility-toughness combination in CrCoNi medium-entropy alloy

**DOI:** 10.1038/ncomms14390

**Published:** 2017-02-20

**Authors:** Zijiao Zhang, Hongwei Sheng, Zhangjie Wang, Bernd Gludovatz, Ze Zhang, Easo P. George, Qian Yu, Scott X. Mao, Robert O. Ritchie

**Affiliations:** 1 Center of Electron Microscopy & State Key Laboratory of Silicon Materials, Department of Materials Science & Engineering, Zhejiang University, Hangzhou 310027, China; 2 Department of Physics and Astronomy, George Mason University, Fairfax, Virginia 22030, USA; 3 Department of Materials Science and Engineering, Xi'an Jiaotong University, Xi'an 710049, China; 4 Materials Sciences Division, Lawrence Berkeley National Laboratory, Berkeley, California 94720, USA; 5 Materials Sciences & Technology Division, Oak Ridge National Laboratory, Oak Ridge, Tennessee 37831, USA; 6 Department of Mechanical Engineering & Materials Science, University of Pittsburgh, Pittsburgh, Pennsylvania 15261, USA; 7 Department of Materials Science & Engineering, University of California, Berkeley, California 94720, USA

## Abstract

Combinations of high strength and ductility are hard to attain in metals. Exceptions include materials exhibiting twinning-induced plasticity. To understand how the strength-ductility trade-off can be defeated, we apply *in situ*, and aberration-corrected scanning, transmission electron microscopy to examine deformation mechanisms in the medium-entropy alloy CrCoNi that exhibits one of the highest combinations of strength, ductility and toughness on record. *Ab initio* modelling suggests that it has negative stacking-fault energy at 0K and high propensity for twinning. With deformation we find that a three-dimensional (3D) hierarchical twin network forms from the activation of three twinning systems. This serves a dual function: conventional twin-boundary (TB) strengthening from blockage of dislocations impinging on TBs, coupled with the 3D twin network which offers pathways for dislocation glide along, and cross-slip between, intersecting TB-matrix interfaces. The stable twin architecture is not disrupted by interfacial dislocation glide, serving as a continuous source of strength, ductility and toughness.

## Abstract (web summary)

Materials that show twinning-induced plasticity can offer unusual combinations of strength and ductility. Here, authors study deformation twinning and dislocation behaviour in a medium-entropy alloy CrCoNi and find a three-dimensional (3D) hierarchical twin network that forms from the activation of three twinning systems.

Strength and ductility (and hence toughness) are key mechanical properties of structural materials^1^,^2^,^3^,^4^,^5^,^6^,^7^,^8^,^9^,^10^,^11^,^12^,^13^,^14^, although these properties are often mutually exclusive^15^. In crystalline metallic materials, strength and ductility depend on the presence of crystal defects and how they move under mechanical loading. Dislocations serve as the prime carrier of plasticity in metals and their motion to create plastic deformation is strongly influenced by interactions with other dislocations as well as defects such as solute atoms^14^,^16^,^17^,^18^,^19^,^20^, stacking faults^21^,^22^, grain boundaries (GBs)^23^,^24^,^25^ and reinforcement particles or precipitates^26^,^27^. In general, strong interactions (obstacles) strengthen a material although this often limits its ability to plastically deform. Twinning, however, appears to be a deformation mechanism capable of defeating this ‘conflict' between strength and ductility^1^,^2^,^3^,^4^,^5^,^6^,^8^,^9^,^16^,^28^,^29^. The presence of twins usually serves to impede dislocation motion and induce strengthening, but multiple twinning systems can also enhance ductility. This is apparent in twinning-induced plasticity (TWIP) steels, which are known to form multiple types of twins that result in high strength with substantial uniform ductility^6^,^10^,^11^,^12^. Another notable example includes certain equiatomic multi-element alloys, termed high-entropy alloys^30^ (HEAs), based on the CrMnFeCoNi composition that have a single-phase face-centered cubic structure (fcc) structure^31^,^32^ with relatively low-stacking-fault energies (SFEs), comparable with 304 stainless steels^33^ and relatively large separations between the Shockley partials^34^. These alloys can display excellent strength^9^,^35^, ductility^9^,^35^ and toughness^1^ at room temperature, properties which are further enhanced or maintained at cryogenic temperatures where deformation nano-twinning becomes a more prominent mode of deformation. Despite our recent observations of various toughening mechanisms occurring on the nanometre length-scale in the vicinity of a crack tip in such an alloy^8^, the quantitative strengthening effect from deformation twinning and the nature of twinning-induced plasticity remain somewhat uncertain.

In this work, we show that the mechanical behaviour of the nominally equiatomic alloy CrCoNi, a medium-entropy alloy (MEA) with a single-phase, fcc structure^16^,^36^, and even better mechanical properties than the CrMnFeCoNi high-entropy alloy—specifically, a high strength (∼1 GPa) and ductility (∼60% uniform elongation) combined with exceptional fracture toughness (*K*_Ic_>200 MPa✓m)^8^,^16^—can be related to its capacity for twinning-controlled deformation. Our *ab initio* calculations based on the density-functional theory (DFT) suggest that this alloy has a negative SFE at 0 K, similar to what has been reported for some other systems^37^,^38^,^39^,^40^,^41^ and high propensity for twinning when compared with pure fcc metals^42^. We seek to demonstrate that a 3D twin network is formed within the grains of this fcc CrCoNi MEA and that the interactions of dislocations with these twins can lead to a simultaneous increase of strength and ductility, that is, to identify the origin of its excellent mechanical properties.

## Results

### Numerical simulations

DFT-based *ab initio* calculations were performed to compute the formation energy of the equiatomic CrCoNi alloy with different crystal structures as well as their planar fault energies. In previous theoretical work^33^,^43^,^44^, the formation energies of medium- and high-entropy alloys were treated with the coherent potential approximation (CPA)^44^,^45^ or the special quasi-random structure (SQS)^46^ method in conjunction with *ab initio* treatments. In this study, the formation energies of CrCoNi crystals were explicitly assessed with the ‘multiple randomly populated supercell' approach. The configurational dependence of the formation energy of CrCoNi MEAs is shown in Supplementary Fig. 1. In our calculations, atoms of the different elements comprising the MEA were randomly assigned to the lattice points of the corresponding crystal structure, followed by geometrical optimization to obtain the ground state configuration of each structure. Although computationally demanding, this approach gives us an advantage by allowing direct evaluation of the stacking-fault energies and their formation energy barriers.

Supplementary Table 1 lists the formation energies of hypothetical face-centered cubic (fcc), hexagonal close-packed (hcp), double hcp (dhcp) and 9R structures for the CrCoNi alloy. Note that their respective stacking sequences are: fcc—…ABC ABC…, hcp—…AB AB…, dhcp—…ABAC … and 9R—…ABA BCB CAC…

It was found that the formation energy of hcp CrCoNi at 0 K is slightly lower than that of fcc CrCoNi, which implies that the hcp structure can be a competing phase during the synthesis of CrCoNi (Supplementary Fig. 1). The energy difference at 0 K between fcc and hcp CrCoNi was found to be ∼7 meV per atom (that is, 0.68 kJ mol^−1^). According to our free-energy calculations based on the quasi-harmonic approximation (Supplementary Fig. 2), the fcc structure gains a free-energy advantage at higher temperatures (for example, 500 K), due mainly to the contribution from lattice vibration. Similar results (namely that hcp is stable at low temperatures and fcc at higher temperatures) were obtained recently for the 5-element HEA, CrMnFeCoNi, when, in addition to configurational entropy, other entropic contributions including electronic, vibrational and magnetic were included in the total energy calculations^44^. Lattice distortions arising from size mismatches of the constituent atoms in the CrCoNi MEA with the fcc or hcp structure were also examined from the atomic pair-distribution functions (PDFs) of CrCoNi crystals; these PDFs of fcc and hcp CrCoNi at 0 K are shown in Supplementary Fig. 3. Further description of the analyses is provided in Supplementary Note 1.

**Figure 1:**
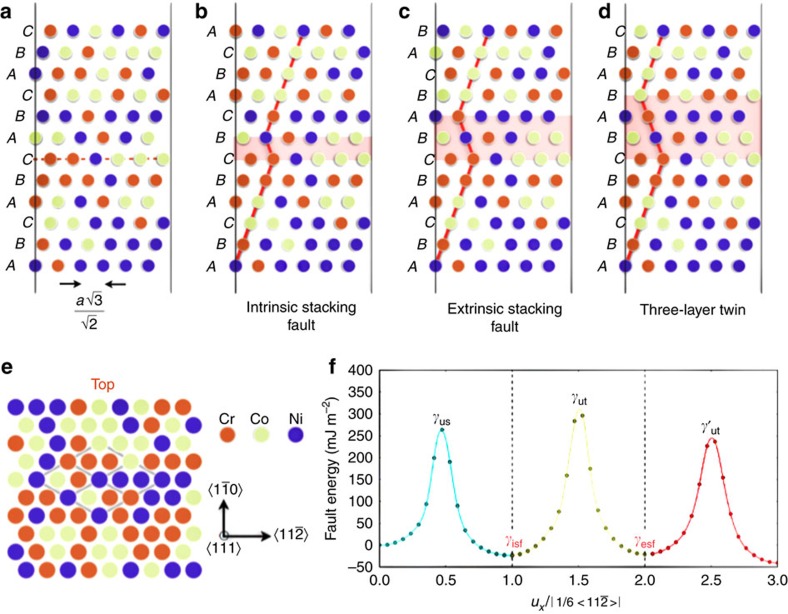
Stacking faults and their *ab initio* calculated energies for the CrCoNi alloy. (**a**–**d**) show the atomic configurations of the original fcc structure, intrinsic stacking fault, two-layer extrinsic fault and three-layer twin, respectively. (**e**) Top view image showing the close-packed (111) plane. (**f**) Energy barriers along the displacement pathway 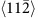 direction in **e** that result in the two types of stacking fault and a twin boundary. The smallest displacement along the pathway 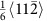 is given by the magnitude of the Burgers vector of a Shockley partial dislocation, *b*_s_. Here, *γ*_isf_ and *γ*_esf_ represent the stacking-fault energies of intrinsic (**b**) and extrinsic (**c**) stacking faults, respectively. *γ*_us_ is the unstable stacking-fault energy, and *γ*_ut_ denotes the energy barrier for the formation of the initial twin boundary. After *γ*_ut_ is overcome, the subsequent energy barriers (for example, 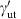) for creating multi-layer twins are smaller than *γ*_ut_. The energy barriers along the displacement pathway were obtained with the nudged-elastic band method in our *ab initio* calculations.

The stacking faults in fcc CrCoNi were analysed with *ab initio* modelling; results are shown in Fig. 1. A slab of CrCoNi with the fcc structure (360 atoms) was first created by sequentially stacking 12 close-packed (111) atomic planes. The slabs have two free surfaces. In the simulation cell, in the direction perpendicular to the slab (*z* direction), a vacuum of thickness 1.6 nm is inserted between slabs to separate each slab from the adjoining ones in the next periodic cell (to minimize the interactions of the slabs). The atomic configurations of the slab were geometrically relaxed in the three directions. Next, an intrinsic stacking fault, of energy γ_isf_, was created by shifting the top six layers along the 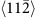 direction by the Burgers vector of the Shockley partial 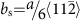, where *a* is the lattice parameter; this resulted in a stacking sequence: ABCABC|BCABCA, shown in Fig. 1b. The stacking-fault energy is obtained by the energy difference of the two optimized structures normalized by the area of the stacking fault *A*.

Subsequent displacement of the top five layers in Fig. 1b along the 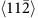 direction results in a two-layer stacking fault (Fig. 1c), which is called an extrinsic stacking fault (ABCABC|B|ABCAB). Further shifting of the fault in Fig. 1c leads to a three-layer fault, shown in Fig. 1d and so on. As such, continued shifting of the crystal will create a twinned structure with a twin boundary. The calculated energies of the intrinsic stacking fault, *γ*_isf_, and twin boundary, *γ*_TB_, are given in Table 1. The energy γ_us_ is that of the unstable stacking fault, and *γ*_ut_ is the nucleation energy barrier for the formation of twin boundaries (Fig. 1f).

Alternatively, stacking-fault energies can be calculated through the use of a parametrized method, that is, the axial interaction model (AIM)^47^. Using AIM, the energy of an intrinsic stacking fault can be expressed in terms of the energies (*E*) of the various structures and the fault areas (*A*) as:


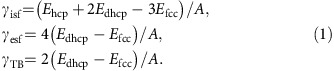


Substituting the values of the formation energies of the hcp, fcc and dhcp structures from Supplementary Table 1, we obtain *γ*_isf_=−23 mJ m^−2^, *γ*_esf_ =−20 mJ m^−2^ and *γ*_TB_=−10 mJ m^−2^, respectively, which are generally consistent with the results of our explicit calculations provided in Table 1.

An energy barrier must be overcome when an intrinsic stacking fault is created by displacing the upper half of the crystal, as demonstrated in Fig. 1f. This energy barrier corresponds to the unstable stacking-fault energy of fcc CrCoNi, *γ*_us_. On further displacement of the crystal with a pre-existing fault (as shown in Fig. 1b), another energy barrier is crossed, namely *γ*_ut_, the energy barrier for twin-boundary formation. These energy barriers lie on the minimum energy paths between the stacking faults, and have profound implications for the deformation mechanism of the crystal.

In this work, we used the nudged-elastic band (NEB) method^48^ in the DFT calculation to obtain the unstable stacking-fault energies along the deformation paths. During the NEB simulation, the atoms are relaxed to the minimum energy paths. It should be mentioned that, without the atomic relaxation process, the unstable fault energy calculated for the rigidly shifted crystal is about 1.5–2 times higher than the correct values shown in Fig. 1f.

Having obtained the stacking-fault energy values in Fig. 1f, we are able to evaluate the propensity of twinning (‘twinnability') in the CrCoNi crystal. On the basis of Tadmor and Bernstein's work on fcc metals^42^,^49^, an empirical expression for twinnability, *τ*_ta_, is:


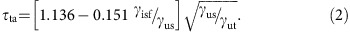


Using this equation, the dimensionless twinnability for the present CrCoNi fcc alloy is *τ*_ta_=1.07±0.02, which is higher than that of all pure fcc metals^42^ (for example, the numerically derived *τ*_ta_ for Ag is ∼1.05), and comparable to that of Fe–Cr–Ni TWIP steels^50^ (1.08–1.12, based on equation (2) and the *ab initio* data from ref. 50). The high propensity for twinning of fcc CrCoNi appears to be a factor in the excellent strength, ductility and toughness of this MEA.

### Nanostructural characterization

This calculated twinnability is consistent with the twinning observed in the CrCoNi alloy after plastic deformation^8^. To examine its specific atomic-scale mechanisms of deformation, we performed advanced structure characterization utilizing quantitative *in situ* transmission electron microscopy (TEM) and aberration-corrected scanning transmission electron microscopy (STEM). We also performed quantitative *in situ* TEM compression tests to investigate the strengthening effect of a twin boundary in the CrCoNi alloy. By compressing micro-pillars with and without a twin boundary, we were able to quantify the strengthening effect from blocked dislocations at a twin boundary (TB). The dislocation-twin and twin–twin interactions during plastic deformation were systematically studied *in situ* in the TEM to reveal the influence of the 3D twin architecture on the plastic deformation of the material. Remarkably, while the twin boundaries contributed to strengthening by acting as barriers to dislocation motion, the interconnected twin boundaries in the 3D twin network can also generate significant ductility by offering multiple pathways for dislocation motion along the twin boundaries.

**Figure 2:**
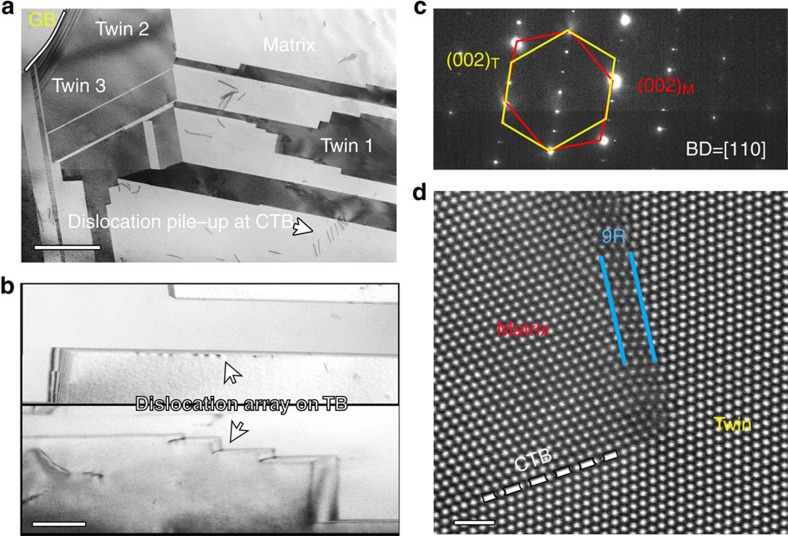
TEM of twin structures in the CrCoNi alloy. (**a**) Bright-field TEM image showing the hierarchical twinning architecture in a grain of the CrCoNi alloy. A grain boundary is marked by the yellow line near the top-left corner, and multiple twinning systems are labelled. Scale bar, 1 μm. (**b**) Low-magnification bright-field TEM image showing dislocation arrays on the twin boundary. Scale bar, 500 nm. (**c**) SAED pattern along <110> beam direction from the region on the CTB circled in blue in **a** showing extra spots which belong to the twin. (**d**) HAADF STEM image showing the structure of a CTB and an ITB which contains a 9R structure. This image was taken from an intersection of CTB and ITB of twin 1 in **a**. Scale bar, 500 pm.

Post-mortem TEM investigations of the CrCoNi samples revealed that large numbers of deformation twinning events occurred in samples deformed in uniaxial tension; three twinning systems were activated within individual coarse grains, forming a 3D twin architecture in which multiple twins with different thicknesses form and intersect each other. The twins typically had thicknesses ranging from ∼100 nm to several micrometres (examples are shown in Fig. 2a), and were present within most grains that we examined. Two twin variants, comprising a compound twin structure that is common in fcc crystals, were observed to intersect each other; these are labelled as ‘Twin 1' and ‘Twin 2' in Fig. 2a. Selected area electron diffraction (SAED) patterns taken along the [110] zone axis (Fig. 2c) reveal separate sets of spots, belonging to the matrix and the twin, respectively. Two types of twin boundaries were observed: 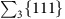 coherent twin boundaries (CTBs) and 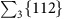 incoherent twin boundaries (ITBs). Dislocations interact with these TBs in various ways. For example, dislocations pile-up at CTBs when they glide on a {111} plane that is not parallel to the CTB, as shown in Fig. 2a. Moreover, dislocation arrays can also form near the TBs, either at a CTB or the intersections of CTBs and ITBs (Fig. 2b). Corresponding atomic structures of a dislocation at the intersection of a CTB {111} and ITB {112} are shown in Fig. 2d, which is a typical high-angle annular dark field (HAADF) STEM image obtained in the aberration-corrected TEAM0.5 TEM microscope operating at 200 kV. A 9R structure composed of a set of Shockley partial dislocations with a repetitive sequence b2:b1:b3 (refs 51, 52, 53, 54) on every (111) plane can be seen in the {112} ITB. The twin architecture is presumably formed due to twin–twin interactions. A smaller twin (for example, Twin 3 in Fig. 2) can be generated within large parent twins by the ITB of other twins (Twin 1) advancing and hitting the CTB of a thick twin (Twin 2) during plastic deformation, as shown in Fig. 2.

### *In situ* mechanical testing

**Figure 3:**
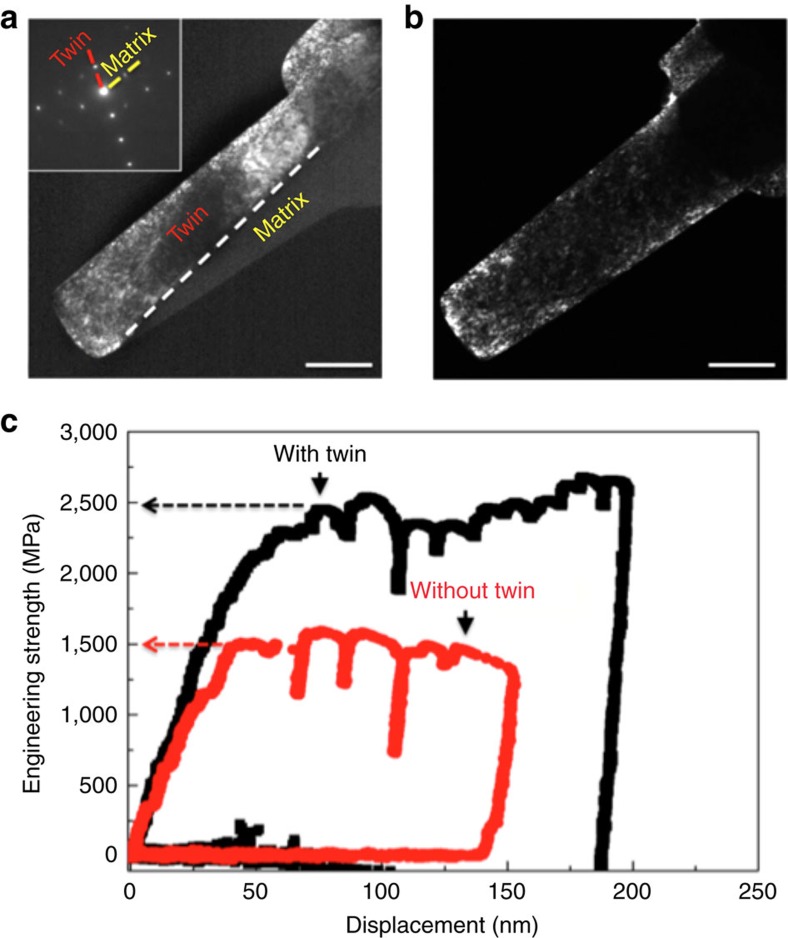
*In situ* compression of CrCoNi micro-pillars with and without a twin boundary. (**a**) Low-magnification dark field TEM image showing the structure of the pillar containing a twin; the inset in **a** shows the SAED pattern of the pillar. The spots from the matrix and twin can be distinguished. The twin boundary is marked by a white dashed line. The zone axis is [110]. Scale bar, 200 nm. (**b**) Low-magnification dark field TEM image showing the pillar with no twin (*g*=[220]). Scale bar, 200 nm. (**c**) Engineering stress-displacement curves of compression tests on the two pillars shown in **a** and **b**. The pillar containing a twin boundary is 67% stronger than the pillar without the boundary.

A high density of twins is expected to have a marked strengthening effect as twin boundaries can act as effective barriers to dislocation motion. Using micro-pillar compression experiments, we attempted to quantify the contribution of an individual twin boundary to the strength of this CrCoNi alloy. We used ∼300-nm diameter, focused-ion beam (FIB) machined micro-pillars, which were tested in a JEOL 3010 TEM at 300 kV, either with or without a {111} twin boundary (and no other boundary), as shown respectively in the dark field images in Fig. 3a,b and in the Supplementary Movie 1. The SAED pattern in the inset of Fig. 3a shows two sets of spots, confirming the existence of the twin. Engineering stress-displacement curves from the compression of these pillars (Fig. 3c) clearly show the much higher strength of the pillar containing an initial twin. Specifically, the maximum strength of the pillar with a pre-existing twin exceeded 2.5 GPa, that is, about 1 GPa higher than that without a twin. As the sizes of the pillars were kept the same to minimize possible size effects, such an elevation in strength can be plausibly attributed to the presence of the twin boundary. However, as these nano-pillar tests only investigate limited volumes, *in situ* TEM straining tests were also performed to systematically investigate the dislocation-twin and twin–twin interactions, with particular focus on the formation of the 3D twin architecture and how this architecture influences the dislocation activity.

**Figure 4:**
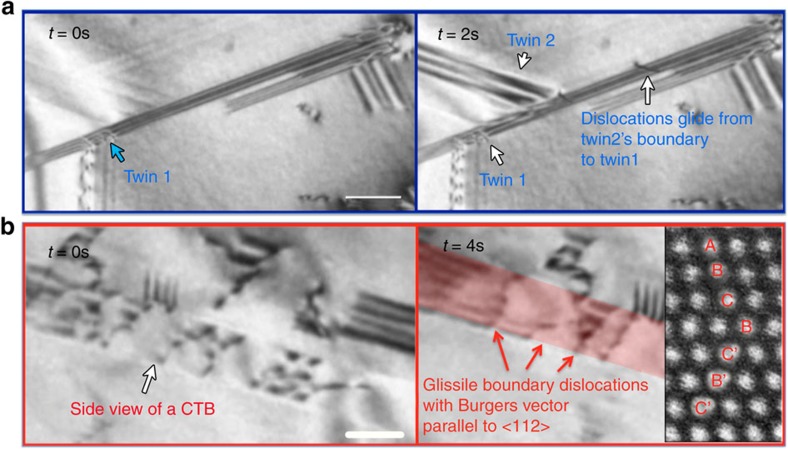
*In situ* imaging shows the dislocation and twin sequences during deformation. (**a**) TEM image sequence showing the formation of the twinning architecture and the dynamic process of dislocations gliding from one twin boundary to another. Scale bar, 200 nm. (**b**) TEM images showing the glide of paired partial dislocations on a CTB. The inset on the right shows the change of stacking at the twin boundary from fcc to hcp due to the glide of a partial dislocation. Scale bar, 200 nm.

The *in situ* straining experiments enabled direct observations of the formation of the twinning architecture and the interactions between dislocations and twin boundaries, as shown in Fig. 4 and Supplementary Movies 2–5. During straining, multiple types of deformation twins were quickly activated, forming a hierarchical architecture consisting of twins with different thicknesses. Figure 4a shows the dynamic formation of two twins, labelled as ‘Twin 1' and ‘Twin 2', and their interaction to form a twin junction. It was observed that the twins propagated very fast until they hit the boundary of other twins. During further straining, the hierarchical twin architecture remains stable with no noticeable thickening or de-twinning. Activity of screw dislocations was also observed at the TB. A dislocation, originally gliding on the boundary between ‘Twin 2' and the matrix, can be seen to impinge at the intersection of ‘Twin 1' and ‘Twin 2', before subsequently cross-slipping onto the boundary between ‘Twin 1' and the matrix, on another {111} plane. From the real-time observations, the twin boundaries clearly act as strong barriers to dislocation motion, preventing dislocations from moving across them and resulting in the pile-up of dislocations (Supplementary Movie 3), as has also been reported previously^55^,^56^,^57^,^58^,^59^. Although impeding dislocation motion usually compromises ductility, the generation of multiple types of deformation twins contributes to the homogeneous plastic deformation. More importantly, the intersected boundaries of the hierarchical twins in the CrCoNi MEA were able to serve as fast channels for dislocation movement. Specifically, dislocations can cross-slip from one twin boundary to another using the ‘overpass' constructed by the intersected twin boundaries, resulting in a more homogeneous distribution of dislocation activity in three dimensions. Although we did not capture the detailed process, sessile dislocation(s) should be involved in the cross-slip phenomenon of those imperfect dislocations at the intersection of the twin planes. We observed that, during further straining, the majority of the deformation inside individual grains occurred through gliding of dislocations along the twin boundaries in the 3D architecture. The dislocations moved fast along the boundaries, which is consistent with previous reports that demonstrated that the dislocation motion on a twin boundary would be faster than that in the matrix since the impediment from the image force is null if the Burgers vector of dislocations is parallel to the twin boundary^41^,^60^,^61^. Figure 4b shows a side view of a twin boundary in which the TBs are inclined and intersect both the top and bottom surfaces of the TEM film. Although cross-slip of partial dislocations^62^,^63^ at twin–twin junctions could not be fully observed due to the limited time-resolution of *in situ* TEM, in Fig. 4b we show the glissile boundary dislocations with Burgers vector parallel to <112> gliding on the twin boundary; however, the twin boundaries remained stable as these dislocations moved along them, that is, there was no evidence of either twin growth or de-twinning occurring during such deformation (Fig. 4b and Supplementary Movies 4 and 5). As such, a high ductility was achieved.

## Discussion

On the basis of the *in situ* straining of the CrCoNi, we have found that a three-dimensional hierarchical twin network is established within individual grains in this MEA, associated with its very low (negative) stacking-fault energy calculated at 0 K; this network presents substantial barriers for dislocation motion, and contributes to its high strength and significant strain hardening. However, at the same time, the network provides multiple pathways for the easy motion of dislocations, which permits the simultaneous generation of significant plastic deformation. Importantly, the formation of the 3D twin network is achieved within relatively large grains (∼5–50 μm in size^8^), and is found to be stable against de-twinning as deformation continues with the twin-boundary interfaces serving as pathways for dislocation slip. This observation is significantly different from that previously reported in twinning-deformation dominated materials^3^,^4^,^6^,^7^,^64^. As a result, high strength coupled with high ductility and continuous strain hardening is achieved in this alloy, which presents the perfect ingredients for its exceptionally high fracture toughness^8^. As lower (cryogenic) temperatures serve to elevate the strength and hence are expected to further promote twinning activity, the damage tolerance (strength with toughness) of this alloy is destined to be enhanced at such low temperatures, which is exactly what has been observed experimentally^8^. This dual effect on dislocation activity is a consequence of the three-dimensional hierarchical twinning architecture that is generated with plastic strain, which in turn results from the high twinnability of this particular alloy.

We note that 3D twinning networks resulting from the activation of three twinning systems may also form within individual grains in TWIP steels, specifically under complex loading conditions such as torsion. The formation of a high density of twin lamellae serves to refine the grains, thereby hindering dislocation motion and generating strain hardening; ductility is considered to originate from the formation of the deformation twins. In comparison, the density of twins in CrCoNi is much lower, but the intersected twin boundaries of different twins act to construct an overpass, which enables fast motion of dislocations on the boundaries and cross-slip from one boundary to another, leading to more homogeneous plastic deformation even within individual grains.

We believe that documentation of the enhanced twinning-induced toughness of this MEA may serve not only to improve our understanding of the extraordinary structural behaviour of specific high-/MEAs but also to present new directions for the future design of metallic alloys with unprecedented combinations of mechanical properties.

## Methods

### Materials preparation and microstructural characterization

Samples for this study were extracted from a previously produced nominally equiatomic CrCoNi MEA whose microstructure and mechanical properties were reported in a recent paper^8^ where details of its processing and mechanical characterization can be found. Atomic structures were investigated using the aberration-corrected TEAM0.5 transmission electron microscope (operating at 200 kV), housed at the National Center for Electron Microscopy at the Lawrence Berkeley National Laboratory (LBNL), and the *in situ* compression tests were performed using a Hysitron PI95 nanoindenter in a JEOL 3010 microscope at 300 KV. The nanopillars for the *in situ* compression tests were produced using focused-ion beam techniques; details of sample preparation and *in situ* compression have been described in previous studies^65^,^66^. The *in situ* TEM tensile tests were conducted at room temperature using a Gatan model 654 single-tilt straining holder in an FEI Tecnai G2 F20 TEM operating at 200 kV. Roughly 12 samples, thinned by jet polishing and well attached to the substrate, were selected for *in situ* tensile straining and detailed TEM investigation as described in a previous paper^22^. Time-resolved TEM and HRTEM images of the regions of interest were recorded with a Gatan CCD camera at 10 frames per second.

### Computational methods

*Ab initio* calculations were performed with the plane-wave based Vienna *Ab initio* Simulation Package (VASP)^67^,^68^. The valence electrons of Cr, Co and Ni are, respectively, 6, 9, 10 in the calculation and the projector augmented wave (PAW) method^69^ was used for the pseudo-potential treatment of core and semi-core electrons. In our calculation, the crystal structures, with periodic boundary conditions, typically contain 300–500 atoms, depending on the specific crystal structure. Atoms are randomly assigned to the lattice points of the corresponding crystal structure, followed by geometrical optimization to obtain the ground state configuration of each structure. The formation energy of each structure corresponds to a statistical mean of 80 such calculations. The Purdew–Wang-type generalized gradient approximation (GGA)^70^ was employed for the exchange-correlation functional. Our calculations were mainly performed on non-magnetic structures (that is, non-spin-polarized), but the effect of magnetization was also analysed with the results presented in Supplementary Fig. 4. The unstable stacking-fault energy and the energy barrier for twinning were analysed with the nudged-elastic band method^48^ implemented in the VASP package. To obtain the formation energy, the initially created lattice structure was subjected to geometrical optimization with high-precision calculations (the energy cutoff used in this work was 337.0 eV) on the Γ point of the Brillouin zone. Geometrical optimization was fully achieved when the total energy converged to within 10^−4^ eV between successive steps. Having optimized the volume while allowing the atoms to relax to their ground state, the formation energy (cohesive energy) was derived by subtracting the sum of single-atom energies from the total energy.

### Data availability

The data that support the findings of this study are available from the corresponding authors upon request.

## Additional information

**How to cite this article:** Zhang, Z. *et al*. Dislocation mechanisms and 3D twin architectures generate exceptional strength-ductility-toughness combination in CrCoNi medium-entropy alloy. *Nat. Commun.* **8,** 14390 doi: 10.1038/ncomms14390 (2017).

**Publisher's note**: Springer Nature remains neutral with regard to jurisdictional claims in published maps and institutional affiliations.

## Supplementary Material

Supplementary Information Supplementary Figures, Supplementary Tables, Supplementary Notes and Supplementary References

Supplementary Movie 1 In situ mechanical testing of a FIBed CrCoNi pillar containing a twin. A pillar with ~300-nm diameter and with a {111} twin boundary compressed in situ under the dark-field imaging mode.

Supplementary Movie 2 Observation of twin-twin interaction and its influence on dislocation glide. Two twins belonging to different twin systems nucleated and formed a junction. A dislocation, which originally glided on the boundary of twin 2, impinged at the intersection of the twins and later cross-slipped onto the boundary of twin 1. A dislocation, which originally glided on the boundary of twin 2, impinged at the intersection of the twins and later cross-slipped onto the boundary of twin 1.

Supplementary Movie 3 Dislocations pile up on the twin boundary. Dislocation motion across the twin boundary is seen to be difficult.

Supplementary Movie 4 Movement of leading and trailing pairs of partial dislocations on the coherent twin boundary plane. During further deformation (~ 8% global strain) in the CrCoNi alloy, partial dislocation pairs glided on the twin boundaries, which served as channels for dislocation movement. Stacking faults between leading and trailing partials are not visible.

Supplementary Movie 5 Additional sequence of the movement of leading and trailing pairs of partial dislocations on the coherent twin boundary plane. Similar observations as in Supplementary Movie 4, except that stacking faults between leading and trailing partials are now visible because of the different diffraction vectors.

## Figures and Tables

**Table 1:** *Ab initio* calculations of various fault energies in CrCoNi MEA at 0 K.

**Fault**	***γ***_**isf**_	***γ***_**us**_	***γ***_**TB**_	***γ***_**ut**_
Fault energy, *E* (mJ m^−2^)	−24	264	−17	310

## References

[b1] Gludovatz B. . A fracture-resistant high-entropy alloy for cryogenic applications. Science 345, 1153–1158 (2014). 25190791 10.1126/science.1254581

[b2] Zhao Y. H. . Simultaneously increasing the ductility and strength of ultra-fine-grained pure copper. Adv. Mater. 18, 2949–2953 (2006).

[b3] Lu K., Lu L. & Suresh S. Strengthening materials by engineering coherent internal boundaries at the nanoscale. Science 324, 349–352 (2009). 19372422 10.1126/science.1159610

[b4] Lu L., Chen X., Huang X. & Lu K. Revealing the maximum strength in nanotwinned copper. Science 323, 607–610 (2009). 19179523 10.1126/science.1167641

[b5] Zhu T., Li J., Samanta A., Kim H. G. & Suresh S. Interfacial plasticity governs strain rate sensitivity and ductility in nanostructured metals. Proc. Natl Acad. Sci. USA 104, 3031–3036 (2007). 17360604 10.1073/pnas.0611097104 PMC1805608

[b6] Wei Y. . Evading the strength-ductility trade-off dilemma in steel through gradient hierarchical nanotwins. Nat. Commun. 5, 3580 (2014). 24686581 10.1038/ncomms4580 PMC3988817

[b7] Kou H., Lu J. & Li Y. High-strength and high-ductility nanostructured and amorphous metallic materials. Adv. Mater. 26, 5518–5524 (2014). 24975572 10.1002/adma.201401595

[b8] Gludovatz B. . Exceptional damage-tolerance of a medium-entropy alloy CrCoNi at cryogenic temperatures. Nat. Commun. 7, 10602 (2016). 26830651 10.1038/ncomms10602 PMC4740901

[b9] Otto F. . The influences of temperature and microstructure on the tensile properties of a CoCrFeMnNi high-entropy alloy. Acta Mater. 61, 5743–5755 (2013).

[b10] Frommeyer G., Brüx U. & Neumann P. Supra-ductile and high-strength manganese-TRIP/TWIP steels for high energy absorption purposes. ISIJ Int. 43, 438–446 (2003).

[b11] Karaman I., Sehitoglu H., Gall K., Chumlyakov Y. & Maier H. Deformation of single crystal Hadfield steel by twinning and slip. Acta Mater. 48, 1345–1359 (2000).

[b12] Karaman I. . Modeling the deformation behavior of Hadfield steel single and polycrystals due to twinning and slip. Acta Mater. 48, 2031–2047 (2000).

[b13] Deng Y. . Design of a twinning-induced plasticity high entropy alloy. Acta Mater. 94, 124–133 (2015).

[b14] Li Z., Pradeep K. G., Deng Y., Raabe D. & Tasan C. C. Metastable high-entropy dual-phase alloys overcome the strength–ductility trade-off. Nature 534, 227–230 (2016). 27279217 10.1038/nature17981

[b15] Ritchie R. O. The conflicts between strength and toughness. Nat. Mater. 10, 817–822 (2011). 22020005 10.1038/nmat3115

[b16] Wu Z., Bei H., Pharr G. M. & George E. P. Temperature dependence of the mechanical properties of equiatomic solid solution alloys with face-centered cubic crystal structures. Acta Mater. 81, 428–441 (2014).

[b17] Romaner L., Ambrosch-Draxl C. & Pippan R. Effect of rhenium on the dislocation core structure in tungsten. Phys. Rev. Lett. 104, 195503 (2010). 20866976 10.1103/PhysRevLett.104.195503

[b18] Leyson G. P., Curtin W. A., Hector L. G. Jr. & Woodward C. F. Quantitative prediction of solute strengthening in aluminium alloys. Nat. Mater. 9, 750–755 (2010). 20676087 10.1038/nmat2813

[b19] Liddicoat P. V. . Nanostructural hierarchy increases the strength of aluminium alloys. Nat. Commun. 1, 63 (2010). 20842199 10.1038/ncomms1062

[b20] Xie K. Y. . The effect of pre-existing defects on the strength and deformation behavior of α-Fe nanopillars. Acta Mater. 61, 439–452 (2013).

[b21] Yu Q., Li S., Minor A. M., Sun J. & Ma E. High-strength titanium alloy nanopillars with stacking faults and enhanced plastic flow. Appl. Phys. Lett. 100, 063109 (2012).

[b22] Zhang Z. . Nanoscale origins of the damage tolerance of the high-entropy alloy CrMnFeCoNi. Nat. Commun. 6, 10143 (2015). 26647978 10.1038/ncomms10143 PMC4682111

[b23] Kumar K., Van Swygenhoven H. & Suresh S. Mechanical behavior of nanocrystalline metals and alloys. Acta Mater. 51, 5743–5774 (2003).

[b24] Chen J., Lu L. & Lu K. Hardness and strain rate sensitivity of nanocrystalline Cu. Scripta Mater. 54, 1913–1918 (2006).

[b25] Li H. & Ebrahimi F. Tensile behavior of a nanocrystalline Ni–Fe alloy. Acta Mater. 54, 2877–2886 (2006).

[b26] Gladman T. Precipitation hardening in metals. Mater. Sci. Technol. 15, 30–36 (1999).

[b27] Zimmerman A., Palumbo G., Aust K. & Erb U. Mechanical properties of nickel silicon carbide nanocomposites. Mate. Sci. Eng. A 328, 137–146 (2002).

[b28] Dao M., Lu L., Shen Y. F. & Suresh S. Strength, strain-rate sensitivity and ductility of copper with nanoscale twins. Acta Mater. 54, 5421–5432 (2006).

[b29] Yan F. K., Liu G. Z., Tao N. R. & Lu K. Strength and ductility of 316L austenitic stainless steel strengthened by nano-scale twin bundles. Acta Mater. 60, 1059–1071 (2012).

[b30] Yeh J. W. . Nanostructured High-entropy alloys with multiple principal elements: novel alloy design concepts and outcomes. Adv. Eng. Mater 6, 299–303 (2004).

[b31] Cantor B., Chang I. T. H., Knight P. & Vincent A. J. B. Microstructural development in equiatomic multicomponent alloys. Mater. Sci. Eng. A 375–377, 213–218 (2004).

[b32] Otto F., Yang Y., Bei H. & George E. P. Relative effects of enthalpy and entropy on the phase stability of equiatomic high-entropy alloys. Acta Mater. 61, 2628–2638 (2013).

[b33] Zaddach A. J., Niu C., Koch C. C. & Irving D. L. Mechanical properties and stacking fault energies of NiFeCrCoMn high-entropy alloy. JOM 65, 1780–1789 (2013).

[b34] Smith T. . Atomic-scale characterization and modeling of 60° dislocations in a high-entropy alloy. Acta Mater. 110, 352–363 (2016).

[b35] Gali A. & George E. P. Tensile properties of high- and medium-entropy alloys. Intermetallics 39, 74–78 (2013).

[b36] Wu Z., Bei H., Otto F., Pharr G. M. & George E. P. Recovery, recrystallization, grain growth and phase stability of a family of FCC-structured multi-component equiatomic solid solution alloys. Intermetallics 46, 131–140 (2014).

[b37] Ferreira P. & Müllner P. A thermodynamic model for the stacking-fault energy. Acta Mater. 46, 4479–4484 (1998).

[b38] Kibey S., Liu J., Curtis M., Johnson D. & Sehitoglu H. Effect of nitrogen on generalized stacking fault energy and stacking fault widths in high nitrogen steels. Acta Mater. 54, 2991–3001 (2006).

[b39] Suzuki H. Segregation of solute atoms to stacking faults. J. Phys. Soc. Jpn 17, 322–325 (1962).

[b40] Schramm R. & Reed R. Stacking fault energies of seven commercial austenitic stainless steels. Metall. Trans. A 6, 1345–1351 (1975).

[b41] Koizumi Y. . Strain-induced martensitic transformation near twin boundaries in a biomedical Co–Cr–Mo alloy with negative stacking fault energy. Acta Mater. 61, 1648–1661 (2013).

[b42] Tadmor E. & Bernstein N. A first-principles measure for the twinnability of FCC metals. J. Mech. Phys. Solids 52, 2507–2519 (2004).

[b43] Niu C. . Spin-driven ordering of Cr in the equiatomic high entropy alloy NiFeCrCo. Appl. Phys. Lett. 106, 161906 (2015).

[b44] Ma D., Grabowski B., Körmann F., Neugebauer J. & Raabe D. *Ab initio* thermodynamics of the CoCrFeMnNi high entropy alloy: importance of entropy contributions beyond the configurational one. Acta Mater. 100, 90–97 (2015).

[b45] Vitos L., Skriver H. L., Johansson B. & Kollár J. Application of the exact muffin-tin orbitals theory: the spherical cell approximation. Comput. Mater. Sci. 18, 24–38 (2000).

[b46] Zunger A., Wei S.-H., Ferreira L. & Bernard J. E. Special quasirandom structures. Phys. Rev. Lett. 65, 353 (1990). 10042897 10.1103/PhysRevLett.65.353

[b47] Vitos L., Korzhavyi P. A., Nilsson J. & Johansson B. Stacking fault energy and magnetism in austenitic stainless steels. Phys. Scr. 77, 065703 (2008).

[b48] Jónsson H., Mills G. & Jacobsen K. W. in *Classical and Quantum Dynamics in Condensed Phase Simulations* (eds Berne, B. J. .) 385 (World Scientific Publ. Co, 1998).

[b49] Bernstein N. & Tadmor E. Tight-binding calculations of stacking energies and twinnability in fcc metals. Phys. Rev. B 69, 094116 (2004).

[b50] Li W. . First-principles prediction of the deformation modes in austenitic Fe–Cr–Ni alloys. Appl. Phys. Lett. 108, 081903 (2016).

[b51] Liu L., Wang J., Gong S. K. & Mao S. X. High resolution transmission electron microscope observation of zero-strain deformation twinning mechanisms in Ag. Phys. Rev. Lett. 106, 175504 (2011). 21635047 10.1103/PhysRevLett.106.175504

[b52] Ernst F. . Theoretical prediction and direct observation of the 9R structure in Ag. Phys. Rev. Lett. 69, 620 (1992). 10046988 10.1103/PhysRevLett.69.620

[b53] Wolf U., Ernst F., Muschik T., Finnis M. & Fischmeister H. The influence of grain boundary inclination on the structure and energy of σ=3 grain boundaries in copper. Philos. Mag. A 66, 991–1016 (1992).

[b54] Schmidt C., Finnis M., Ernst F. & Vitek V. Theoretical and experimental investigations of structures and energies of Σ=3,[112] tilt grain boundaries in copper. Philos. Mag. A 77, 1161–1184 (1998).

[b55] Rohatgi A., Vecchio K. S. & Gray G. T. III The influence of stacking fault energy on the mechanical behavior of Cu and Cu-Al alloys: Deformation twinning, work hardening, and dynamic recovery. Metall. Mater. Trans. A 32, 135–145 (2001).

[b56] Afanasyev K. A. & Sansoz F. Strengthening in gold nanopillars with nanoscale twins. Nano Lett. 7, 2056–2062 (2007).

[b57] Zheng Y., Lu J., Zhang H. & Chen Z. Strengthening and toughening by interface-mediated slip transfer reaction in nanotwinned copper. Scripta Mater. 60, 508–511 (2009).

[b58] Zhu L., Qu S., Guo X. & Lu J. Analysis of the twin spacing and grain size effects on mechanical properties in hierarchically nanotwinned face-centered cubic metals based on a mechanism-based plasticity model. J. Mech. Phys. Solids 76, 162–179 (2015).

[b59] Coujou A. Déformation *in situ* d'un alliage à basse énergie de faute d'empilement. Acta Metall. 31, 1505–1515 (1983).

[b60] Priester L. Grain Boundaries: From Theory to Engineering Vol. 172, Springer Science & Business Media (2012).

[b61] Priester L. & Khalfallah O. Image force on a lattice dislocation due to a grain boundary in anisotropic fcc materials. Philos. Mag. A 69, 471–484 (1994).

[b62] Itakura M., Kaburaki H., Yamaguchi M. & Tsuru T. Novel Cross-slip mechanism of pyramidal screw dislocations in magnesium. Phys. Rev. Lett. 116, 225501 (2016). 27314728 10.1103/PhysRevLett.116.225501

[b63] Vanderschaeve G. Cross-slip of partial dislocations via the stair rod mode. The zigzag propagation of deformation microtwins in ordering alloys. Phys. Status Sol. (A) 100, 59–68 (1987).

[b64] Shen Y. F., Lu L., Lu Q. H., Jin Z. H. & Lu K. Tensile properties of copper with nano-scale twins. Scripta Mater. 52, 989–994 (2005).

[b65] Yu Q. . Origin of dramatic oxygen solute strengthening effect in titanium. Science 347, 635–639 (2015). 25657243 10.1126/science.1260485

[b66] Shan Z., Mishra R. K., Asif S. S., Warren O. L. & Minor A. M. Mechanical annealing and source-limited deformation in submicrometre-diameter Ni crystals. Nat. Mater. 7, 115–119 (2008). 18157134 10.1038/nmat2085

[b67] Kresse G. & Hafner J. *Ab initio* molecular dynamics for liquid metals. Phys. Rev. B 47, 558 (1993). 10.1103/physrevb.47.558 10004490

[b68] Kresse G. & Hafner J. *Ab initio* molecular-dynamics simulation of the liquid-metal–amorphous-semiconductor transition in germanium. Phys. Rev. B 49, 14251 (1994). 10.1103/physrevb.49.14251 10010505

[b69] Kresse G. & Joubert D. From ultrasoft pseudopotentials to the projector augmented-wave method. Phys. Rev. B 59, 1758 (1999).

[b70] Perdew J. P. . Atoms, molecules, solids, and surfaces: applications of the generalized gradient approximation for exchange and correlation. Phys. Rev. B 46, 6671 (1992). 10.1103/physrevb.46.6671 10002368

