# Supplementary material for: Dislocation mechanisms and 3D twin architectures generate exceptional strength-ductility-toughness combination in CrCoNi medium-entropy alloy
Source: Nat Commun. 2017 Feb 20;8:14390. doi: 10.1038/ncomms14390 (PMC5321736; doi:10.1038/ncomms14390)
Supplement: Supplementary Information — Supplementary Figures, Supplementary Tables, Supplementary Notes and Supplementary References [file ncomms14390-s1.pdf]

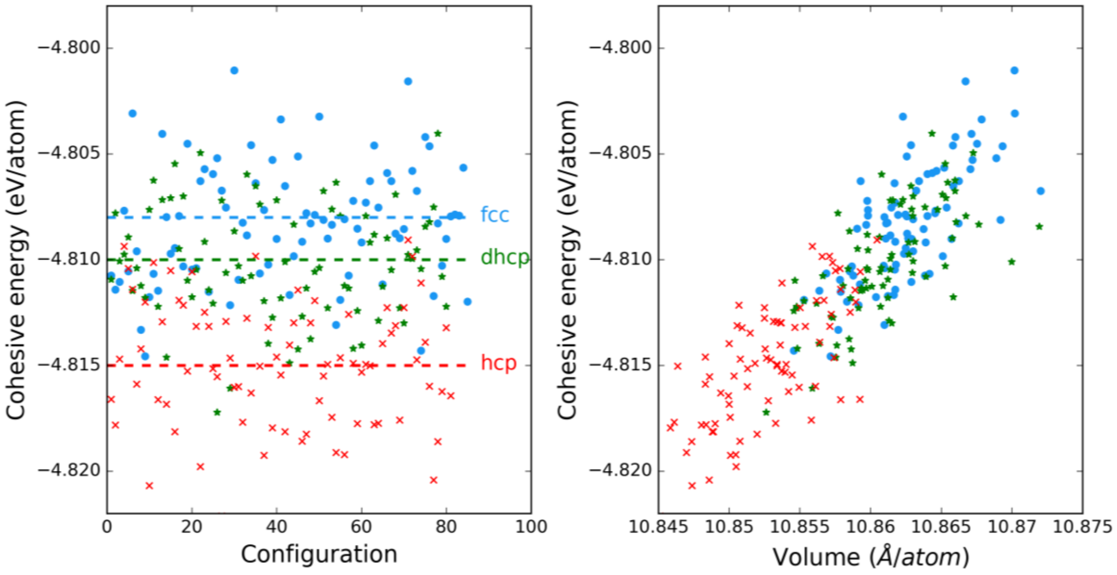

Supplementary Figure 1. Configurational and volume dependence of the formation (cohesive) energy of face-centered cubic (fcc), hexagonal close packed (hcp) and double hcp (dhcp) CrCoNi alloys. The formation energies were evaluated with the "multiple randomly populated supercell" method, which show a relatively wide distribution. The average energy differences between the phases are very small (a few milli-electron volts), which calls for careful scrutiny of approximations used in other *ab initio* approaches of evaluating the stacking fault energy, such as the coherent potential approximation (CPA) and special quasi-random structure (SQS) methods.

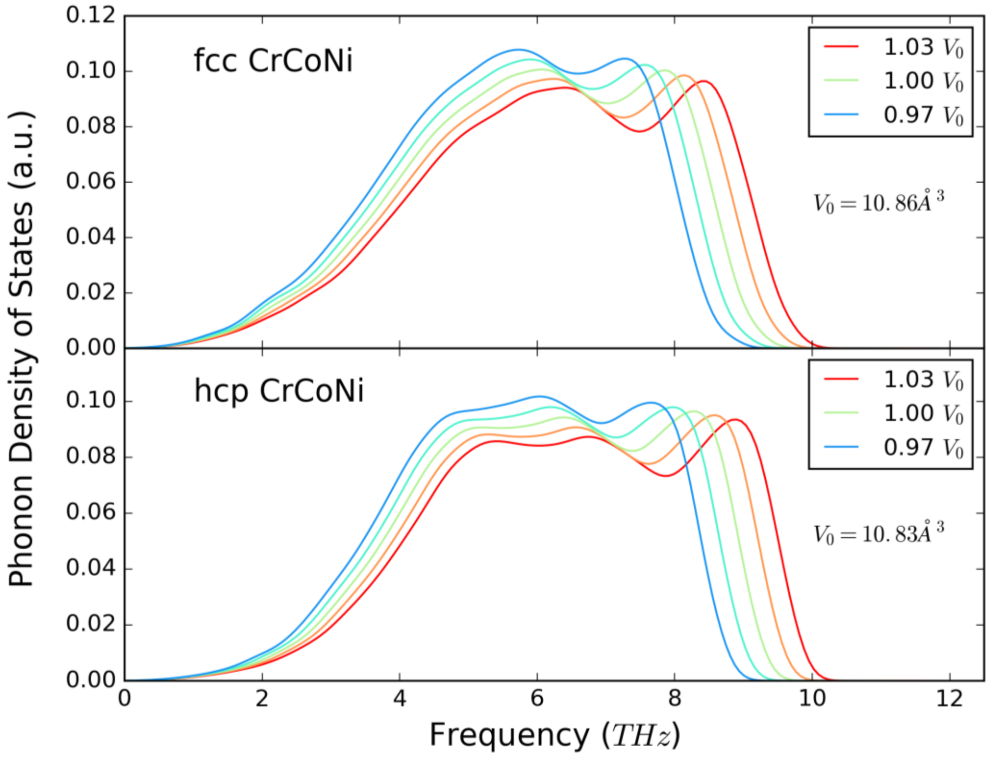

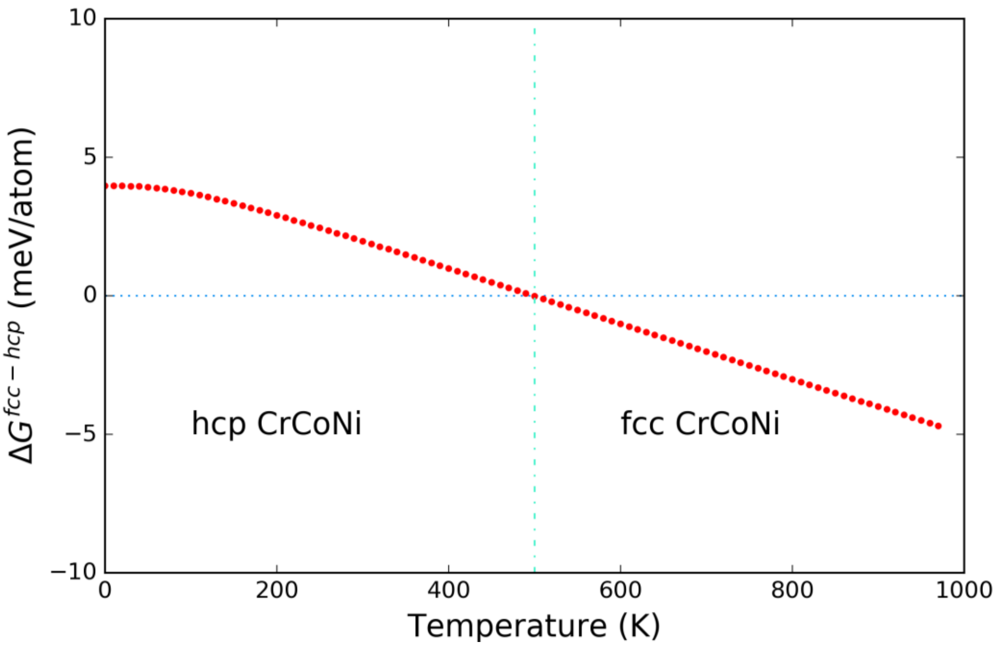

Supplementary Figure 2. Lattice vibration contribution to the structural stability of fcc CrCoNi. (Top) Phonon density of states of fcc and hcp CrCoNi for different unit cell volumes. (Bottom) Gibbs free energy difference between fcc and hcp CrCoNi structures, manifesting the contribution of lattice dynamics in stabilizing fcc CrCoNi at higher temperatures.

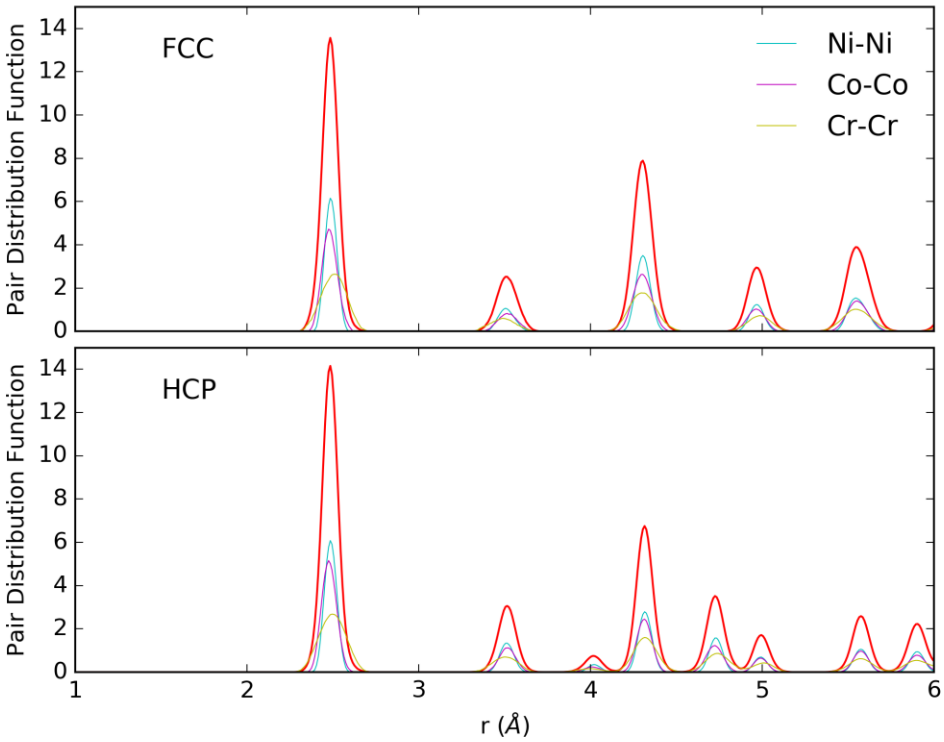

Supplementary Figure 3. Pair distribution functions of the CrCoNi alloy obtained from *ab initio* modeling. Atomic pair distribution functions (PDFs) of the CrCoNi medium-entropy alloy with fcc and hcp structures at the ground state (red curves), showing slight lattice distortions (broad peaks) resulting from the different atomic sizes of the constituent elements. The PDF is defined as $g(r) = \frac{1}{4\pi r^2}\frac{1}{N\rho}\sum_{i-1}^{N}\sum_{k\neq i}^{N}\langle\delta(r-|r_k-r_i|)\rangle$, where N is the number of particles and ρ is the number density. The peak broadening seen in the $g(r)$ function reflects the degree of static lattice distortion of the CrCoNi alloy. Also shown in the figure are the partial PDFs of the constituent atoms (Ni-Ni, Co-Co, and Cr-Cr), from which the average bond-distances of different atomic pairs can be evaluated. The degree of lattice distortion is relatively small in the CrCoNi alloy, ~2.5% of the average interatomic distance.

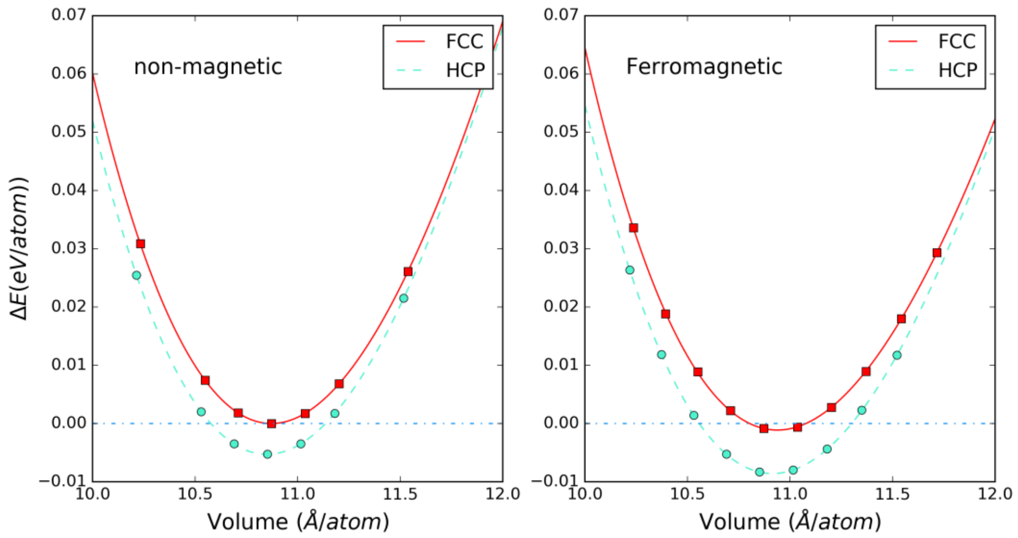

Supplementary Figure 4. Results of *ab initio* calculations of the energy differences between fcc and hcp CrCoNi at 0K. Equation of states of fcc and hcp CrCoNi phases in the non-magnetic (left panel) and the ferromagnetic (right panel) states. The energies are plotted with reference to the non-magnetic fcc CrCoNi structure at the ground state. In both cases, the hcp structure is found to have a smaller formation energy. The magnetic contribution is found to be small in the CrCoNi alloy.

Supplementary Table

Supplementary Table 1. Formation energies of the fcc, hcp, dhcp and 9R structures in CrCoNi.

Crystal structure	Cohesive energy (eV)	Lattice parameter (Å)	Lattice distortion δ
**fcc**	-4.808 ± 0.0006	a = 3.516	2.5%
**hcp**	-4.815 ± 0.0006	a = 2.499, c = 4.013	2.3%
**dhcp**	-4.810 ± 0.0005	—	—
**9R**	-4.809 ± 0.0010	—	—

Supplementary Note 1

First-principles analysis of the CrCoNi alloy

Contribution of lattice dynamics to the phase stability of CrCoNi. Experimentally, fcc CrCoNi is the thermodynamically stable phase at room temperature. Here we demonstrate that hcp is the stable structure for this alloy at 0 K and that, as the temperature is raised, lattice vibrations contribute to fcc becoming more stable than hcp.

We used the quasi-harmonic approximation (QHA)[1] to evaluate the stability of the non-magnetic fcc and hcp phases of CrCoNi. To test the validity of the QHA method for treating phonons, we ran first-principles molecular dynamics simulation and obtained the total phonon spectra of fcc CrCoNi through the Fourier transformation of the velocity auto-correlation functions[1]. Our results suggest that lattice anharmonicity is insignificant in the CrCoNi alloys when the temperature is below 500K.

The Gibbs free energy at zero pressure in the QHA is given by: $G(T) = \min_V[U(V) + E_{zp}(V) - TS(T,V)]$, where $U(V)$ is the formation energy as a function of volume V; $E_{zp}(V)$ is the zero-point energy and $S(T,V)$ is the sum of the configurational and vibrational entropies. Both E_{zp} and S are related to lattice vibration of the crystals, which can be computationally derived in first-principles treatments. In evaluating the Gibbs free energy $G(T)$, we neglected magnetic contributions. In this work, lattice vibration frequencies and phonon density of states of CrCoNi were calculated using the frozen-phonon method to obtain the dynamical matrices. The calculation was performed using the PHONOPY computational code[2] interfaced with the Vienna *Ab-initio* Simulation Package[3,4]. Having obtained the vibrational density of states, the Gibbs free energy difference between the fcc and hcp phases of CrCoNi was obtained, as shown in the bottom panel of Supplementary Figure 2. Our results show that lattice vibration helps stabilize fcc CrCoNi which has a lower Gibbs free energy above a temperature of ~500K. Experimentally, the fcc

structure is found to be stable also at lower temperatures (~300 K) suggesting that there are other factors, beyond those considered here, that affect phase stability.

Numerical estimates of lattice distortions of fcc and hcp CrCoNi . Lattice distortion in the CrCoNi medium-entropy alloy arising from size mismatches of the constituent atoms can be seen from their atomic pair-distribution functions (PDFs)[5]. Supplementary Figure 3 shows the PDFs of fcc and hcp CrCoNi at 0K. The peak broadening indicates that the bond distances of the atomic pairs have a distribution. For the first nearest neighbor shell, it was found that the average Cr-Cr bond distance is slightly larger than those of Ni-Ni and Co-Co ($r_{Cr\text{-}Cr}$ = 0.2505 nm, $r_{Ni\text{-}Ni}$ = 0.2489 nm, $r_{Co\text{-}Co}$ = 0.2481 nm). To quantify the degree of lattice distortion, we used the root mean square displacement (RMSD) of the atoms from the perfect lattice sites: $< \mu_s > = \sqrt{\{[r_i - r_o]^2\}}$, where r_i is the atomic position, and r_o is the position of perfect crystal lattice site corresponding to atom i; <…> is the ensemble average. The static lattice distortions, $< \mu_s >$, for fcc CrCoNi and hcp CrCoNi are calculated to be 0.063 Å and 0.056 Å, respectively. The normalized lattice distortion, $\delta = < \mu_s > / \bar{r}$, is listed in Table I, where $\bar{r}$ is the average bond-distance 2.485 Å.

Magnetic contribution to the formation energy of fcc and hcp CrCoNi. In the non-magnetic *ab initio* calculation presented in the text, it is found that the formation energy at 0 K of hcp CrCoNi is slightly more negative than fcc CrCoNi, implying that the stacking fault energy of CrCoNi (γ_{sf}) is negative based on the AIM apporach[6]. Here we consider the magnetic contribution to the formation energy of CrCoNi by comparing the energetics of ferromagnetic (FM) and non-magnetic (NM) states of CrCoNi.

Both fcc and hcp CrCoNi structures have been analyzed with collinear spin-polarized DFT calculations following the computational method stated in the main text, and the results are shown in Supplementary Figure 4. It can be seen that ferromagnetic CrCoNi still has a lower energy than non-magnetic CrCoNi, indicating that at low temperatures CrCoNi assumes a ferromagnetic state. However, we

conclude that the magnetic contribution to the formation energy is negligibly small. In the ferromagnetic state, the energy difference between fcc and hcp phases is about 7.4 meV/atom, which is only slightly larger than the energy difference between nonmagnetic fcc and hcp CrCoNi (~5 meV).

In a recent experiment[7], it was reported that the ferromagnetic transition temperature of the CrCoNi alloy is below 2 K, which is in line with our focus on the paramagnetic state of CrCoNi in this work. We have conducted explicit DFT calculations to evaluate the local magnetic moments of Cr, Co and Ni in the CrCoNi alloys in the paramagnetic state. To this end, we employed the disordered local moment (DLM) model implemented in the EMTO-CPA package[6] and calculated the local magnetic moments of Co, Ni, Cr in fcc and hcp CrCoNi alloys. Our results clearly indicate that all the local moments of the atomic species are essentially zero. A similar phenomenon has also been reported in the CrCoFeMnNi high-entropy alloy[8], where the local magnetic moments of Cr, Co, and Ni were found to be zero. We conclude that the magnetic contribution of CrCoNi can be ignored within the current research scope.

Supplementary References

1. Dove, M.T., *Introduction to Lattice Dynamics*, Cambridge University Press (1993).
2. Togo, A. & Tanaka, I., First principles phonon calculations in materials science, *Scripta Mater.* **108**, 1 (2015).
3. Kresse G. & Hafner, J. *Ab initio* molecular dynamics for liquid metals. *Phys. Rev. B* **47**, 558 (1993).
4. Kresse G. & Hafner, J. *Ab initio* molecular-dynamics simulation of the liquid-metal-amorphous-semiconductor transition in germanium. *Phys. Rev. B* **49**, 14251 (1994).
5. Chandler, D., *Introduction to Modern Statistical Mechanics*. Oxford University Press, Oxford, U.K. (1987).
6. Vitos, L., Skriver, H., Johansson, B. & Kollar, J. Application of the exact Muffin-tin orbitals theory: The spherical cell approximation, *Comp. Mater. Sci.* **18**, 24 (2000)
7. Sales, B.C., *et al.*, Quantum critical behavior in a concentrated ternary solid solution, *Scientific Reports* **6**, 26179 (2016).

8. Ma, D., *et al.*, *Ab initio* thermodynamics of the CoCrFeMnNi high entropy alloy: Importance of entropy contributions beyond the configurational one, *Acta Mater*. **100**, 90 (2015).
